# Oxidative DNA Damage Mediated by Intranuclear MMP Activity Is Associated with Neuronal Apoptosis in Ischemic Stroke

**DOI:** 10.1155/2016/6927328

**Published:** 2016-01-27

**Authors:** Shihoko Kimura-Ohba, Yi Yang

**Affiliations:** Department of Neurology, School of Medicine, University of New Mexico Health Sciences Center, Albuquerque, NM 87131, USA

## Abstract

Evidence of the pathological roles of matrix metalloproteinases (MMPs) in various neurological disorders has made them attractive therapeutic targets. MMPs disrupt the blood-brain barrier and cause neuronal death and neuroinflammation in acute cerebral ischemia and are critical for angiogenesis during recovery. However, some challenges have to be overcome before MMPs can be further validated as drug targets in stroke injury. Identifying* in vivo* substrates of MMPs should greatly improve our understanding of the mechanisms of ischemic injury and is critical for providing more precise drug targets. Recent works have uncovered nontraditional roles for MMPs in the cytosol and nucleus. These have shed light on intracellular targets and biological actions of MMPs, adding additional layers of complexity for therapeutic MMP inhibition. In this review, we discussed the recent advances made in understanding nuclear location of MMPs, their regulation of intranuclear sorting, and their intranuclear proteolytic activity and substrates. In particular, we highlighted the roles of intranuclear MMPs in oxidative DNA damage, neuronal apoptosis, and neuroinflammation at an early stage of stroke insult. These novel data point to new putative MMP-mediated intranuclear actions in stroke-induced pathological processes and may lead to novel approaches to treatment of stroke and other neurological diseases.

## 1. Introduction

*MMP Family Biology*. Matrix metalloproteinases (MMPs) are proteolytic enzymes that cleave almost all components of the extracellular matrix (ECM) including fibronectin, laminin, proteoglycans, type IV collagen, and tight junction proteins (TJP) and have been related to blood-brain barrier (BBB) opening and neurodegeneration associated with ischemia and neuroinflammation [1–4]. MMPs typically possess signal peptide (predomain) which target the enzyme to the endoplasmic reticulum at the amino terminal and transport it out of the cell. Prodomain containing cysteine switch which maintains its latency is connected to the predomain. There is the hemopexin-like domain that regulates substrate recognition at the carboxy terminal end and which is connected to the catalytic zinc site by a flexible hinge region. The MMPs are divided into several groups according to their domain composition and their ability to degrade individual components of ECM [5]. Matrilysins (MMP-7 and MMP-26) are the smallest members of the family that lack the hemopexin-like domain. Collagenases (MMP-1, MMP-8, and MMP-13) and stromelysins (MMP-3, MMP-10, and MMP-11) are composed of a catalytic domain and a hemopexin-like domain. Gelatinases (MMP-2 and MMP-9) have a compact collagen binding domain called fibronectin-like domain within the catalytic domain. Membrane type MMPs (MT-MMP) (MMP-14, MMP-15, MMP-16, MMP-17, MMP-24, and MMP-25) are anchored to the plasma membrane by a transmembrane domain with a cytoplasmic domain or a glycosylphosphatidylinositol (GPI). Many of the MMPs are specifically regulated at the level of gene expression, but their production as inactive proenzymes is another important level of functional regulation [6]. Most MMPs are not constitutively expressed with a few exceptions (i.e., MMP-2 and MT1-MMP) but are transcribed after cell activation [7]. MMPs are synthesized as enzymatically inactive zymogens (pro-MMPs) and are activated by the “cysteine switch,” which disrupts the interaction between a cysteine in the prodomain and the Zinc ion in the active site [8]. 

*Extracellular and Intracellular Location of MMPs in Stroke*. One of the most studied pathological conditions upon MMPs is hypoxia/ischemia. Several MMPs are involved in degrading ECM and tight junction proteins (TJPs) leading to the disruption of BBB, in both early and late opening of BBB [9, 10]. MMP-2 and MT1-MMP (MMP-14) are present in normal condition in latent or proforms in both human and experimental animals [7]. Constitutive expression of MMP-2 in normal pathological conditions provides an ongoing, well-controlled remodeling of the ECM. MMP-2 in normal condition is found in astrocytic endfeet and cerebrospinal fluid. MMP-2 remains in the pro or latent form until activated by a molecular cascade that involves a trimolecular complex composed of MMP-2, tissue inhibitors of metalloproteinases- (TIMP-) 2, and MT1-MMP. This reaction occurs close to the cell surface where it provides local proteolysis without involvement of the surrounding tissues [11]. The local proteolysis of MMPs leads to the reversible initial opening of BBB induced by stroke insult, which occurs several hours after the onset of reperfusion [9, 10]. This early event in the molecular cascade of hypoxia/ischemia is closely related to an activator of MT1-MMP which is required for the activation of MMP-2, by hypoxia inducible factor- 1*α* (HIF-1*α*) [12]. On the other hand, MMP-3 and MMP-9 are the main inducible MMPs involved in the subacute (24–72 hours after the onset) event of hypoxia/ischemia. This later phase is related to delayed secondary BBB opening during a neuroinflammatory response [10]. Several types of cells are involved in expressing these inducible MMPs. The brain vascular endothelial cells, infiltrating neutrophils and microglia, are the major source of MMP-9 [9, 13, 14]. Induced MMP-3 usually colocalizes with macrophages and microglia. Pericytes express both MMP-3 and MMP-9. Neurons and reactive astrocytes also express MMP-2, MMP-3, and MMP-9 [9, 15–17]. Other MMPs involved in injury cascades include MMP-8 and MMP-13 [18]. Infiltrating neutrophils are the source of MMP-8 [19], and MMP-8 plays a pivotal role in neuroinflammation by disrupting BBB and modulating tumor necrosis factor-*α* (TNF-*α*) activation [20, 21].

Although initially thought to act primarily on extracellular targets in neurovascular unit, mainly on proteins in the basal lamina and TJPs, recent studies have revealed that MMPs are also localized in various intracellular sites, including the nucleus [16, 18, 22–27]. Both MMP-2 and MMP-9 were shown to locate in the Golgi and trans-Golgi network both in nonreactive and in reactive astrocytes [28]. The novel subspecialized and compartmentalized nuclear MMPs may participate in many physiological and pathological cellular processes, in which they can act as both constitutive, regulatory, and inducible proteinases [26]. Intranuclear MMP-2 and MMP-9 were found to contribute to the early nuclear gelatinase activity, which produced cleavage of poly-ADP-ribose polymerase- (PARP-) 1 in ischemic rat brain leading to a decrease of PARP-1 activity, promoting neuronal accumulation of oxidative DNA at the early stage of hypoxia/ischemia [16]. The observations and mechanistic insight are highly novel and demonstrate a significant shift in the MMP activity paradigm in physiological and pathological processes.

## 2. Intranuclear Location of MMPs in Neurons and Nuclear Translocation Mechanism

### 2.1. Intranuclear Location of MMPs in Neurons and Other Cells Involved in Brain Injury

Since 1995, several MMPs have been demonstrated as active proteases localized in nuclei of various human and animal cell types; they are MMP-1, MMP-2, MMP-3, MMP-9, MMP-10, MMP-13, MT1-MMP, and MT-MMP-2 (MMP-15) [26]. MMP-2, MMP-3, MMP-9, MMP-13, and MT1-MMP have all been identified in neuronal nuclei and gelatinases (MMP-2 and MMP-9) and MMP-3 have been implicated in the progression of infarct volume and neuronal death in animal models of stroke and ischemic stroke patients [9, 16, 18, 23, 29–33].

Since the first paper showing gelatinolytic activity in neuronal nuclei in SOD1−/− mice after transient focal cerebral ischemia, several studies showed the localization of gelatinases in neuronal nuclei [29]. Activation of MMP-2 and MMP-9 in the neuronal nuclei has been demonstrated in the brain of rats subjected to transient middle cerebral artery occlusion (MCAO) [16, 23, 29, 34, 35] during acute reperfusion. This increase of nuclear gelatinolytic activity of MMP-2 and/or MMP-9 was also observed in human brain after stroke [16, 18, 33]. Beside the active MMP-2 and MMP-9 in ischemic neuronal nuclei at 3 h reperfusion, increased MT1-MMP and hypoxia-enhanced expression of the proprotein convertase furin were also detected in neuronal nuclei [16]. Furin is an activator of MT1-MMP which is required for the activation of MMP-2 [12]. Since MMP-2 is activated by the trimolecular complex, the nuclear location makes MT1-MMP critical in intranuclear activation of MMP-2 [10]. MMP-13 has been found in activated form within the cell nuclei after cerebral ischemia, in both rats and humans [18]. Immunohistochemistry also revealed that MMP-13 was mainly produced by neurons, in both species, but also by oligodendrocytes in rats, and by astrocytes in humans. This increase of nuclear MMP-13 was also reproduced* in vitro* in rat cortical neuron cultures exposed to oxygen and glucose deprivation (OGD).

The increase in intranuclear gelatinase activation in neurons and endothelial cells is also observed in spontaneous intracerebral hemorrhage (ICH) during acute and chronic hypertension in mice brain [27, 36]. These intranuclear MMPs are also reported in rats' oligodendrocytes (MMP-13; hypoxia/ischemia) and astrocytes both in mice and in humans (gelatinases; both normal and reactive astrocytes) [18, 28, 33]. Colocalization of MMP-2 and TIMP-1 in nuclei was shown in neurons and endothelial cells [37].

### 2.2. Nuclear Translocation Mechanism

In most cases, proteins enter the nucleus through the nuclear pore via a mechanism involving recognition of nuclear location signals (NLSs) by transporter proteins [38]. These signals are recognized by soluble receptors that mediate macromolecular transport through the nuclear pore complex. Two types of human NLSs exist, classical basic Lys-rich and M9-type, which are recognized by importins/karyopherins-*α* and transportin (importin/karyopherin-*β*2), respectively [39]. Sequence analysis of mouse MMP-2 using the PSORT software detected three putative nuclear localization signals (NLS) at positions 98, 115, and 114: two closely positioned four-residue patterns (pat4: RKPR and RKPK), the latter also identified as a seven-residue pattern (pat7: PRKPKWD) [25, 31]. In cell lines and human liver tissue, MMP-3 has been described in cell nuclei [31] and a functionally established pat7 NLS has been identified; alignments of a portion of the mouse MMP-3 and MMP-2 N-terminal sequences indicate that the pat7 NLS positions are nearly identical in both proteins and downstream of the propeptide cleavage sites [22, 25, 28]. Besides MMP-2 and MMP-3, putative NLSs in the sequences of MMP-1, MMP-8, MMP-10, MMP-13, MMP-19, MMP-20, and MMP-23 and MT1-MMP, MT3-MMP, MT4-MMP, and MT5-MMP were also detected [31], which suggests that nuclear entry may be a feature of many MMPs. MMPs that do not contain an NLS may enter the nucleus by binding to cargoes, such as RAN binding proteins, other proteins with an NLS, various types of RNA, and complexes of RNA plus proteins [39]. No typical NLS were identified in the entire mouse MMP-9 sequence that may enter the nucleus bound to the nuclear protein Ku [25, 40].

## 3. Intranuclear MMPs in Neurons Contribute to Cell Apoptosis in Stroke

### 3.1. Intranuclear MMPs, Oxidative DNA Damage, and Apoptosis in Ischemic Neurons

MMPs have been linked to BBB opening and neurodegeneration associated with ischemic stroke [2–4, 9, 35, 41–43]. Then, what would be the function and mechanisms of nuclear MMPs involved in the pathological processes of ischemia and reperfusion injury? Increased gelatinase activity (MMP-2 and MMP-9) in nuclei was implied in a mouse model of transient global cerebral ischemia and associated with delayed neuronal death in hippocampus [35]. Neuronal apoptosis following ischemic injury has been proposed as a mechanism of cell death [43]. Treatment with MMP inhibitors blocks cell death in transient cerebral ischemic stroke [30, 35]. Studies have implicated involvement of MMP activation in oxidative stress and cell apoptosis in cerebral ischemic brains [27, 29, 44]. MMP-2 activity has been considered as an early and key event in oxidative stress-related injury to the heart after ischemia-reperfusion [24]. Oxidative stress can promote the increase of MMP-2 and MMP-9 activities [45, 46]. In addition to triggering DNA damage, oxidative stress activates MMP-2 without proteolytic removal of the propeptide, resulting in an active, full-length MMP-2 [24, 47]. The identification of intranuclear MMP activity in neurons enhances the links between MMPs and oxidative DNA damage in neurons and apoptotic neuronal death after stroke injury.

Oxidative DNA damage and decreased DNA base excision repair function in injured neurons occur as early as 30 minutes after onset of reperfusion in cerebral ischemia [48–52]. MMPs in neuronal nuclei can become activated as early as 15–30 min after the onset of reperfusion, increasing gradually with the progression of reperfusion in MCAO rat brains [23, 29]. A marker of oxidative DNA damage, 8-hydroxy-2′-deoxyguanosine (8-OHdG), has been observed 3–6 h after reperfusion in mice [53]. Furthermore, elevation of 8-OHdG was detected and co-localized with nuclear gelatinases in ischemic neurons at 3 h reperfusion. During treatment with MMP inhibitor before MCAO onset, oxidative DNA, including apurinic/apyrimidinic (AP) sites and 8-OhdG, are significantly attenuated in rat brain tissue at 3 h reperfusion [16] and significantly reduced neuronal apoptosis in ischemic hemispheres at 48 h [54]. The colocalization of nuclear active MMPs with 8-OHdG was also seen in neurons and endothelial cells in a mouse model of intracerebral hemorrhage (ICH), which are associated with, and may precede, spontaneous ICH during hypertension [27, 36]. Thereby, the early increase in intranuclear MMP activity in ischemic neurons suggests a proteolytic role for DNA repair proteins which is associated with neuronal apoptotic death after cerebral ischemia.

### 3.2. DNA Base Excision Repair (BER)

**Figure 1:**
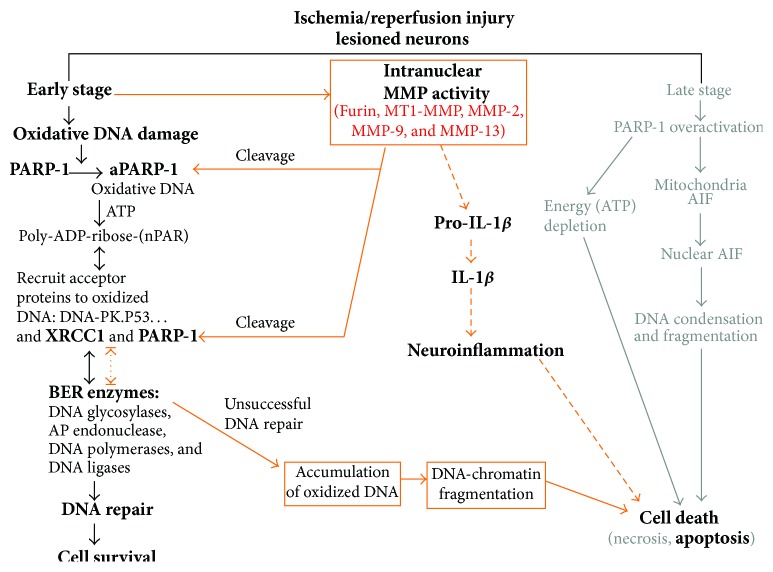
Schematic drawing of hypothesis on how intranuclear MMPs facilitate the oxidative DNA damage and inflammatory cytokines in neurons maturation after ischemic insult. At an early stage after ischemic injury and/or reperfusion, activated nuclear MMPs cleave nuclear proteins PARP-1 and XRCC1, which are critical enzymes in BER pathway for DNA repair and cell suvival. The degradation of these nuclear BER enzymes via MMP-2, activated by furin-enhanced MT1-MMP activity, and MMP-9 during ischemic insult interferes with the DNA repair and enhanced nuclear accumulation of oxidative DNA damage, promoting the ischemic neurons to apoptosis. Intranuclear IL-1*β*, which rapidly elevates as early as 1 h of stroke, is colocalized with intranucler MMP-2 in neurons, suggesting that MMP-2 may contribute to IL-1*β* production early after the beginning of reperfusion. Modified from Yang et al. [16].

Oxidative DNA damage in injured neurons is a prominent event occurring during the early stages after cerebral ischemia [51, 52, 55]. The BER pathway (Figure 1) is the main mechanism in mammalian neuronal nuclei used to repair various types of oxidative DNA damage [52]. BER has 4 main steps (base removal, apurinic/apyrimidinic (AP) site incision, synthesis, and ligation) and involves 4 major classes of DNA repair enzymes: DNA glycosylases, apurinic/apyrimidinic endonucleases (APEs), DNA polymerases (DNA pols), and DNA ligases. Mammalian PARP-1 is a nuclear chromatin-associated multifunctional enzyme [56]. Oxidative DNA damage induces PARP-1 activity which then plays an important role in repair of oxidized DNA and cell survival [57, 58]. When PARP-1 is activated by oxidative DNA damage-related strand breaks, it cleaves NAD^(+)^ into nicotinamide and ADP-ribose and uses the latter to synthesize long branching poly-ADP-ribosylation (PAR) polymers covalently attached to acceptor proteins (including histones, DNA repair enzymes, transcription factors, and PARP-1 itself). The X-ray repair cross-complementing (XRCC) 1 plays a central role in the DNA BER pathway, as it serves as a scaffolding protein interacting with DNA ligase III, DNA pol*β*, APE1, and PARP-1 [59]. During BER and single-strand break repair, XRCC1 coordinates the initial and late stages of BER and is critical for the accurate repair of damaged bases and AP sites [59–62]. XRCC1, DNA ligase III, and DNA pol*β* are established partners of PARP-1 [63–65]. The activation of PARP-1 by DNA breaks triggers the recruitment of XRCC1 to the damage site [63, 66, 67]. Changes in these BER enzymes have been observed in rat brains exposed to cerebral ischemia [51, 65, 68–70].

### 3.3. DNA Repair Enzymes in Hypoxia/Ischemia

PAR formation increases during brain ischemia and participates in stroke pathogenesis and furthermore, inhibitors of PARP are neuroprotective in a model of MCAO [57, 71–73]. Several mechanisms (Figure 1) could underlie neurotoxic actions of PARP-1 in ischemic brain injury [72, 74]. Overactivation of PARP-1 caused by excessive DNA damage leads to cellular energy failure [71, 74]. PAR can trigger mitochondrial release of apoptosis-inducing factor [75] and thereby trigger neuronal death [76, 77]. In addition, PARP-1 has an essential role in expression of NF-*κ*B-dependent genes (including MMP-9) induced by ischemia/reperfusion [78–81]. On the other hand, PARP-1 could act as a survival factor through its capacity to efficiently repair damaged DNA [56]. Abolishing PARP-1 activity in primary cortical neurons can either enhance or prevent apoptotic death, depending on the intensity of oxidative stress. In mild progressive damage that occurs in neurodegenerative diseases, PARP-1 activation plays a neuroprotective role and may contribute to cellular recovery. Thus, mild oxidative stress led to PARP-1-dependent neuroprotection [82] and inhibition of PARP-1 enhanced the vulnerability of neurons to apoptosis following sublethal transient global ischemia [83].

### 3.4. Intranuclear MMP Activity Cleaves PARP-1 and XRCC1, Interfering with Oxidative DNA Repair

The presence of activated MMPs in various intracellular compartments, including nucleus, strongly suggested that MMPs may be responsible for proteolytic actions on substrates within cells [39]. Intracellular substrate proteolysis by MMPs is involved in pathology of cardiac, neurological, protein conformational, and autoimmune diseases. An earlier study showed the intranuclear localization of MMP-2 within cardiac myocytes, which demonstrated an ability to cleave PARP as a substrate* in vitro* [22]. This is the first characterization of potential nuclear substrates for intranuclear MMP-2 proteolysis. In central nervous system (CNS), the nuclear MMP-2 and -9 induced by ischemic injury also demonstrate the same proteolytic activity to PARP-1* in vivo* and* in vitro *[16]. Nuclear gelatinolytic activity colocalized with PARP staining in ischemic brains at 3 h reperfusion and their* in vivo* degradation during ischemia is reduced by the MMP inhibitor. The proteolytic cleavage of PARP-1 by intranuclear and synthesized MMP-2 and MMP-9 yielded product around 43 kDa* in vivo* and* in vitro*, consistent with the early report of MMP-2 in cardiac myocytes [23]. The degradation of PARP-1 by MMP-2 and MMP-9 is independent of the PARP-1 cleavage by caspase-3, where it is well known that PARP-1 cleavage by caspase 3 yields 89 and 24 kDa fragments during apoptosis [16]. Study also showed inhibition of MMP-2 by PARP inhibitors, suggesting that the neuroprotective effects of some PARP inhibitors in ischemic injury may include inhibition of MMP-2 activity [84]. Treatment with an MMP inhibitor significantly reduced the degradation of PARP-1 and attenuated accumulation of oxidative DNA damage in ischemic rat brain tissue [16].

After transient focal ischemia, XRCC1 levels are shown to be markedly reduced in the striatum at 10 minutes after reperfusion, and it is further decreased in the entire middle cerebral artery territory at 1 h, remaining reduced until 24 h of reperfusion [85]. Increased colocalization of nuclear gelatinolytic activity with XRCC1 is seen in ischemic brains at 3 h reperfusion. The reduction of XRCC1 is also detected in the nuclear extracts from ischemic rat brain, which can be reversed by MMP inhibitors* in vivo* [16]. Coincubation with MMP inhibitor inhibited the XRCC1 cleavage caused by recombinant rat MMP-2, murine MMP-9, and gelatinase extracts prepared from nuclear fractions of ischemic rat brain. These results strongly suggest the first time that the early nuclear gelatinolytic proteolysis is involved in the early reduction of XRCC1 induced by transient focal cerebral ischemia [16].

Furthermore, in an OGD model of stroke with primary rat cortical neurons, increased intranuclear location of gelatinase activity and elevated levels of both MMP-2 and MMP-9 of nuclear extracts are detected. A marked decrease of protein level in PARP1, XRCC1, and 8-oxoguanine glycosylase (OGG) 1 and decreased PARP1 activity are presented. Pretreatment of neurons with selective MMP-2/9 inhibitor II significantly decreased gelatinase activity and downregulation of DNA repair enzymes, decreased accumulation of oxidative DNA damage, and promoted neuronal survival after OGD. Importantly, in order to exclude the involvement of gelatinases in neuronal culture medium, which act extracellularly* in vivo* and influence neuronal survival after ischemia/reperfusion, culture medium following OGD and 24 h after reoxygenation was assayed for gelatinase proteolytic activity. OGD or normoxic control treatment with or without MMP inhibitor produced no significant change in extracellular levels of MMP-2 or MMP-9, suggesting a lack of involvement of extracellular gelatinase activity in neuronal apoptosis 24 h reoxygenation [54]. The results suggest a major role for proteolysis by nuclear gelatinases in the induction of an intrinsic neuronal apoptosis pathway independent of MMP-related extracellular matrix proteolysis (Figure 1).

## 4. Intranuclear MMP Activity in Neurons Is Associated with Neuroinflammation

Neuroinflammation contributes to the pathophysiology of cerebral ischemia. At an early stage of stroke, proinflammatory cytokines, such as TNF-*α*, interleukin-1*β* (IL-1*β*), IL-6, and IL-18, are released by activated cells including neurons, astrocytes, microglia, and endothelial cells. Acute elevation of TNF-*α* and IL-1*β* and consequent activation of the IL-1 receptor 1 are harmful to the injured cerebral tissue during ischemic stroke. Cytokines, such as TNF-*α* and IL-1*β*, induce MMP-3 and MMP-9 at the transcriptional level, which is important in the neuroinflammatory response under acute and chronic conditions [10]. In addition to regulation by proinflammatory cytokines, both intracellular catalytic and noncatalytic actions of MMPs, such as MMP-7, also contribute to inflammatory response, leading to cell death [39]. In CNS, microglial activation and inflammation are associated with progressive neuronal apoptosis in human neurodegenerative disorders [86]. Active MMP-3 released by dopaminergic neurons undergoing apoptosis triggers microglial activation and production of proinflammatory factors, including TNF-*α*, IL-6, and IL-1*β*. The MMP-3-mediated activation of microglia is a characteristic response to neuronal apoptosis for inflammatory reactions, which subsequently exacerbate neuronal apoptosis [15, 87].

MMP-2 and MMP-9 have been suggested to process pro-IL-1*β* into bioactive IL-1*β in vitro *[88] and* in vivo* [34, 89]. Studies have proposed that MMPs may mediate neuronal death by proapoptotic signaling upstream of caspases, potentiating neuroinflammation through processing of IL-1*β* in ischemic cortex [87, 90, 91]. Gelatinolytic activity was significantly increased in the nuclei of ischemic core regions, as early as 15 min after reperfusion following MCAo, increasing gradually with the progression of reperfusion [23]. The intracellular MMP proteolytic activity could be one of the earliest pathological events triggered downstream of oxidative stress and inflammation. Recently, study has demonstrated a significant increase of cortical IL-1*β* as early as 1 h after the beginning of reperfusion, the cytokine being mainly expressed in cortical neurons and pericallosal astroglial cells [92]. Importantly, this early increased IL-1*β*, more specifically in neuronal nuclei, colocalizes with elevated intracellular gelatinolytic activity, mainly MMP-2. It is suggested that MMP-2 may contribute to IL-1*β* production early after the beginning of reperfusion and is a possible neuronal source of inflammatory cytokines dependent on intranuclear MMP activation [92]. A recent study evaluated the temporal and spatial evolution of microglia and MMP activation after focal cerebral ischemia in a mouse model of transient MCAO [93]. This study suggests that, at 7 days after MCAO, MMP-9 expression was found in cells, including neurons, microglia, astrocytes, and endothelial cells, with fragmented nuclei, suggesting an associated role in apoptotic processes or to cells undergoing secondary necrosis.

## 5. Summary

MMPs, as proteases that act in the extracellular matrix, are mainly known for their role in promoting cell death, BBB damage, and neuroinflammation at acute stage after cerebral stroke. We and others have demonstrated the intracellular location of different MMPs, including the intranuclear gelatinases (MMP-2 and MMP-9), which are associated with a variety of physiological and pathological processes. In neuronal cells, the nuclear gelatinolytic activity is correlated with the repair pathway of oxidative DNA damage and inflammatory response to ischemic injury. There is strong evidence that the nuclear MMP activity contributes to the apoptotic process and neuroinflammatory cascades at an early stage after ischemic injury. The early detrimental roles of both extracellular and intranuclear MMP activity after stroke make early MMP inhibition a very promising therapeutic approach for neuroprotection. In contrast to acute harmful roles, MMPs participate in neurogenesis as part of the repair process after stroke insult [17, 94, 95]. Study demonstrated that long-term use of MMP inhibitors 7 days or more after stroke onset can lead to increased brain damage characterized by reductions in neurons and newly formed blood vessels [95]. Single-dose of minocycline treatment immediately after reperfusion onset effectively reduces brain injury in rats subjected to transient focal cerebral ischemia due to inhibition on MMP-2/9-mediated occludin degradation and attenuation of caspase dependent and independent apoptotic pathways [96]. Furthermore, this short-term treatment of minocycline at acute stage of stroke also promotes BBB remodeling and alternative microglia/macrophage activation during recovery [97]. While long-term use of MMP inhibitors may lead to side effects, short-term use in acute stroke seems reasonable to test clinically, since the preclinical studies are promising. Minocycline is being tested in preventing the changes in vascular diseases and stroke treatment. It may be worth testing short-term use of minocycline in acute stroke followed by other agents during recovery to reduce its potential detrimental effects on neurovascular remodeling in stroke.

Although studies indicate that the intranuclear MMPs may be involved in a variety of physiological and pathological processes, the mechanisms of nuclear translocation of the different MMPs are not fully characterized. A recent study indicated that MMP-2 is primarily involved in regulation of the activity of stem/progenitor cells that rise to new granule neurons, whereas MMP-9 facilitates migration of the progeny of these cells by proteolysis of extracellular matrix proteins. In this study, MMP-2 expression was found mainly in Sox2-immunopositive stem/progenitor cells, both quiescent and mitotically active, and was localized in both the cytoplasmic compartment and the nucleus [98]. The MMP signaling cascades are becoming even more complex as it is increasingly clear that MMPs can also degrade proteins in the cytoplasm, mitochondria, and nucleus. The success of approach that MMP inhibition as a therapeutic target for the treatment of stroke and other neurological disorders requires a full understanding of the biological processes in each particular disease condition to identify the crucial MMP targets that have to be inhibited [26]. In addition, further research is needed to develop potent and selective agents to avoid the side effects of nonselective MMP inhibitors [8, 99].
